# In vivo genome editing using the Cpf1 ortholog derived from *Eubacterium eligens*

**DOI:** 10.1038/s41598-019-50423-6

**Published:** 2019-09-26

**Authors:** Woo-Chan Ahn, Kwang-Hyun Park, In Seon Bak, Hyung-Nam Song, Yan An, Su-Jin Lee, Mira Jung, Kyeong-Won Yoo, Dae-Yeul Yu, Yong-Sam Kim, Byung-Ha Oh, Eui-Jeon Woo

**Affiliations:** 1 Disease Target Structure Research Center, Korea Research Institute of Bioscience and Biotechnology (KRIBB), Daejeon, 305-806 Republic of Korea; 2 Department of Biological Sciences, KAIST Institute for the Biocentury, Korea Advanced Institute of Science and Technology, Daejeon, 305-701 Republic of Korea; 3 Genome Engineering Laboratory, GHBio Inc, Daejeon, 305-806 Republic of Korea; 4 Genome Editing Research Center, Korea Research Institute of Bioscience and Biotechnology, (KRIBB), Daejeon, 305-806 Republic of Korea; 5 Department of Analytical Bioscience, University of Science and Technology (UST), Daejeon, 305-333 Republic of Korea

**Keywords:** Genetic engineering, Genetic engineering

## Abstract

Cpf1 is an RNA-guided endonuclease that can be programmed to cleave DNA targets. Specific features, such as containing a short crRNA, creating a staggered cleavage pattern and having a low off-target rate, render Cpf1 a promising gene-editing tool. Here, we present a new Cpf1 ortholog, EeCpf1, as a genome-editing tool; this ortholog is derived from the gut bacterial species *Eubacterium eligens*. EeCpf1 exhibits a higher cleavage activity with the Mn^2+^ metal cofactor and efficiently cuts the target DNA with an engineered, nucleotide extended crRNA at the 5′ target site. When mouse blastocysts were injected with multitargeting crRNAs against the IL2R-γ gene, an essential gene for immunodeficient mouse model production, EeCpf1 efficiently generated IL2R-γ knockout mice. For the first time, these results demonstrate that EeCpf1 can be used as an *in vivo* gene-editing tool for the production of knockout mice. The utilization of engineered crRNA with multiple target sites will help to explore the *in vivo* DNA cleavage activities of Cpf1 orthologs from other species that have not been demonstrated.

## Introduction

Clustered regularly interspaced short palindromic repeats (CRISPR) and CRISPR-associated (Cas) systems eliminate invading genetic elements in prokaryotes^1^. CRISPR/Cas effector proteins are RNA-guided endonucleases that can be programmed to cleave DNA or/and RNA targets. Based on the cas gene content and the mechanism of action, CRISPR/Cas systems are classified into two classes and six types. The multiprotein complex of the class 1 systems detects foreign nucleic acids and then degrades DNA or/and RNA^2,3^. Class 2 systems of Cas9 (type II) and Cpf1 (type V) consist of single-component effector proteins that have been repurposed for genome editing in various organisms^4,5^. Cas9 is guided by a hybrid of CRISPR RNA (crRNA) and a trans-activating crRNA (tracrRNA) to cleave the target DNA with a protospacer adjacent motif (PAM). SpCas9, derived from *Streptococcus pyogenes*, was the first CRISPR/Cas protein to enable targeted mutagenesis and is still the most widely used genome-editing tool^6,7^. Recently, the type V of Cpf1 has also been rapidly characterized and developed into a range of genome editing and regulation tools^8^. Some features of the Cpf1 system differ from those of the Cas9 system, although their target recognition mechanism is similar. While the Cas9-RNA complex contains two RNA molecules, Cpf1 is guided by a single crRNA (~41 nt). The PAM site of Cas9 is located 3′ to the target DNA, while in Cpf1, it is located 5′ to the target DNA. Cas9 typically uses a guanine-rich PAM, such as NGG, while Cpf1 utilizes a thymidine-rich PAM, such as TTTN. Cas9 makes a blunt cut adjacent to the PAM, while Cpf1 generates a 5 base pair (bp) staggered cut 17 nt downstream of the PAM^9^. These specific features of Cpf1, together with the feature of lower off-target cleavage rates compared to those of Cas9, can broaden the spectrum of genome editing to various fields^10^. In search of Cpf1 orthologs capable of genome editing, a variety of Cpf1 proteins was explored by the PSI-BLAST program, and 52 nonredundant CRISPR-Cpf1 loci were previously identified^8,11,12^. Among them, seven Cpf1 orthologs (*Francisella novicida* U112 (FnCpf1), *Acidaminococcus sp*. BV36L (AsCpf1), *Lachnospiraceae bacterium* ND2006 (LbCpf1), *Thiomicrospira sp*. Xs5 (TsCpf1), *Moraxella bovoculi* AAX08_00205 (Mb2Cpf1), *Moraxella bovoculi* AAX11_00205 (Mb3Cpf1), and *Butyrivibrio sp*. NC3005 (BsCpf1)) were shown to have DNA cleavage functions *in vivo*, while the Cpf1 variants of *Fn*Cpf1, *As*Cpf1 and *Lb*Cpf1 have been studied most intensively and have been used as gene-editing tools^13,14^. Although EeCpf1 from *Eubacterium eligens* was first identified by its sequence homology, its *in vivo* DNA cleavage activity has never been reported. Recently, we found that the catalytic mutant of EeCpf1 functions as an efficient transcriptional regulator for gene expression in bacteria. Here, we show the *in vivo* DNA cleavage activity of EeCpf1 for the first time and present it as an efficient genome-editing tool.

## Materials and Methods

### Cloning, protein expression and purification

The gene encoding Cpf1 (WP_012739647.1) was amplified from the genomic DNA of *E*. *eligens* (ATCC 27750) by PCR and was ligated into a modified pET-22b(+) plasmid to produce the protein with a 6xHis-tag and a Cysteine Protease Domain (CPD) tag at the C-terminus. The resulting pET22b_EeCpf1-CPD plasmid was transformed into the *E*. *coli* strain BL21-Codon Plus (DE3)-RIL (Agilent Technologies). *E*. *coli* that harbored EeCpf1-CPD were cultured in LB medium that contained ampicillin to an OD600 of 0.6 and were induced by adding 1 mM IPTG at an incubation temperature of 18 °C for 16 hours. The cells were collected by centrifugation (6000 g, 30 min), resuspended in 300 mL of lysis buffer (30 mM Tris-HCl (pH 7.5), 150 mM NaCl, 5 mM β-mercaptoethanol, and 10% glycerol), and disrupted by sonication in an ice bath (VC-600 sonicator; Sonics & Materials). The supernatant was purified by centrifugation (10000 g, 30 min, 4 °C), and the protein was purified using the HisTrap HP, Heparin HP, and Superdex 200 pg columns (GE Healthcare) with an AKTA FPLC system (GE Healthcare) and elution buffer (30 mM Tris-HCl (pH 7.5), 150 mM NaCl, 5 mM β-mercaptoethanol, and 10% glycerol). The C-terminal 6xHis-tag and CPD tag were cleaved with a 200 μM phytic acid treatment^15^. The EedCpf1 mutant that contained the D880A substitution (pET22b-EedCpf1-CPD) was generated with a site-directed mutagenesis kit (Enzynomics) and was purified in the same way as the wild-type protein. The sequences of all bacterial expression plasmids can be found in Supplementary Table 1.

### *In vitro* transcription of crRNAs

The targeting sequence consisted of 24 nucleotides, followed by an ‘TTTN’ sequence called the protospacer adjacent motif (PAM). For *in vitro* transcription, the template DNAs were amplified with the overlap PCR method. The amplified template DNA was purified with a commercial gel extraction kit (Bioneer). *In vitro* transcription was conducted with the purified DNA template using the MEGAshortscript T7 Transcription Kit (Invitrogen) according to the manufacturer’s instructions. The synthesized crRNAs were purified by ethanol precipitation.

### *In vitro* nuclease activity assays

To determine the nuclease activity that targeted the pUC19 plasmid, purified EeCpf1 or EedCpf1 (160 nM) and crRNA (7.6 μM) were incubated at 37 °C for 5 min in reaction buffer (30 mM Tris-HCl (pH 7.5), 100 mM NaCl) with 1 mM MnCl_2_. The reaction was initiated by the addition of the pUC19 plasmid (200 ng) and was incubated at 37 °C for 20 min. The reaction was quenched by the addition of proteinase K (Enzynomics) and incubated at 37 °C for 10 min. All samples were analyzed on a 1% agarose gel. For the *in vitro* activity assay toward the IL2R-γ sequence, the region that contained four target sequences in the IL2R-γ gene was amplified by PCR. The amplified product was purified using a gel extraction kit and was used as a substrate for the IL2R-γ sequence targeting assay. The experiment was conducted following the same process as that of the nuclease activity assay with the pUC19 plasmid, except the amplified substrate was used instead of the plasmid.

### Generation of mutant mice by injection of the EeCpf1/crRNA mixture

The care, use, and treatment of all mice in this study were in strict agreement with the Korean Ministry of Food and Drug Safety (MFDS) guidelines. Protocols were reviewed and approved by the Institutional Animal Care and Use Committee of the Korea Research Institute of Bioscience (KRIBB). Female C57BL/6J mice (6 weeks of age) were superovulated by intraperitoneal injection with 5 IU pregnant mare serum gonadotropin (PSMG, Sigma), followed 46 hours later by an injection of 5 IU human chorionic gonadotropin (hCG, Sigma). Immediately after the hCG injection, female mice were mated 1:1 with male mice (12 weeks of age) of the same strain with proven fertility. The animals were sacrificed 14 hours after hCG administration, and the oviducts were collected. The oocyte-cumulus complexes were released from the oviducts, and the embryos were transferred to microinjection dishes that contained M2 medium (Sigma) under mineral oil. The EeCpf1/crRNA reagent mixture was prepared by dilution of the components into distilled water to obtain the following concentrations: 0.6 µM EeCpf1 protein and 6.1 µM IL2R-γ crRNAs. The reagent mixture was introduced into the cytoplasm of the embryos by microinjection. The injected embryos were cultured in M16 medium (Sigma) under mineral oil. The surviving two-cell stage embryos were surgically implanted into the oviducts of pseudopregnant females.

### Genomic sequence analysis

For PCR amplification, the embryos were lysed in 10 µl of blastocyst lysis buffer (100 mM Tris-HCl (pH 8.3), 100 mM KCl, 0.02% gelatin, 0.45% Tween 20, 10 mg/µl yeast tRNA and 20 mg/ml proteinase K). The samples were incubated at 56 °C for 10 min followed by 95 °C for 10 min and then stored at −4 °C. Four microliters of the crude samples was subjected to PCR amplification. The changes in the genomic DNA sequences of the blastocysts were analyzed by Sanger sequencing analysis (Bioneer, Korea) of a PCR fragment that was amplified from the IL2R-γ gene (primers used: FR, 5′-CAGCTCTTCAGGAACCCTACCAGTTTC-3′ and RP, 5′-CCCCCCCTTAACTGTTTAACCTCAGTC-3′).

### Selection and analysis of off-target sites

Potential off-target sites were selected using Cas-OFFinder (http://www.rgenome.net/Cas-Offinder) with a criterion of less than two bulges and mismatches. On-target and potential off-target sites were amplified by nested PCR. Whether candidate off-target sites were mutated was determined using a T7EI digestion assay and Sanger sequencing.

## Results

### Characterization of the CRISPR/Cas system in *Eubacterium eligens*

**Figure 1:**
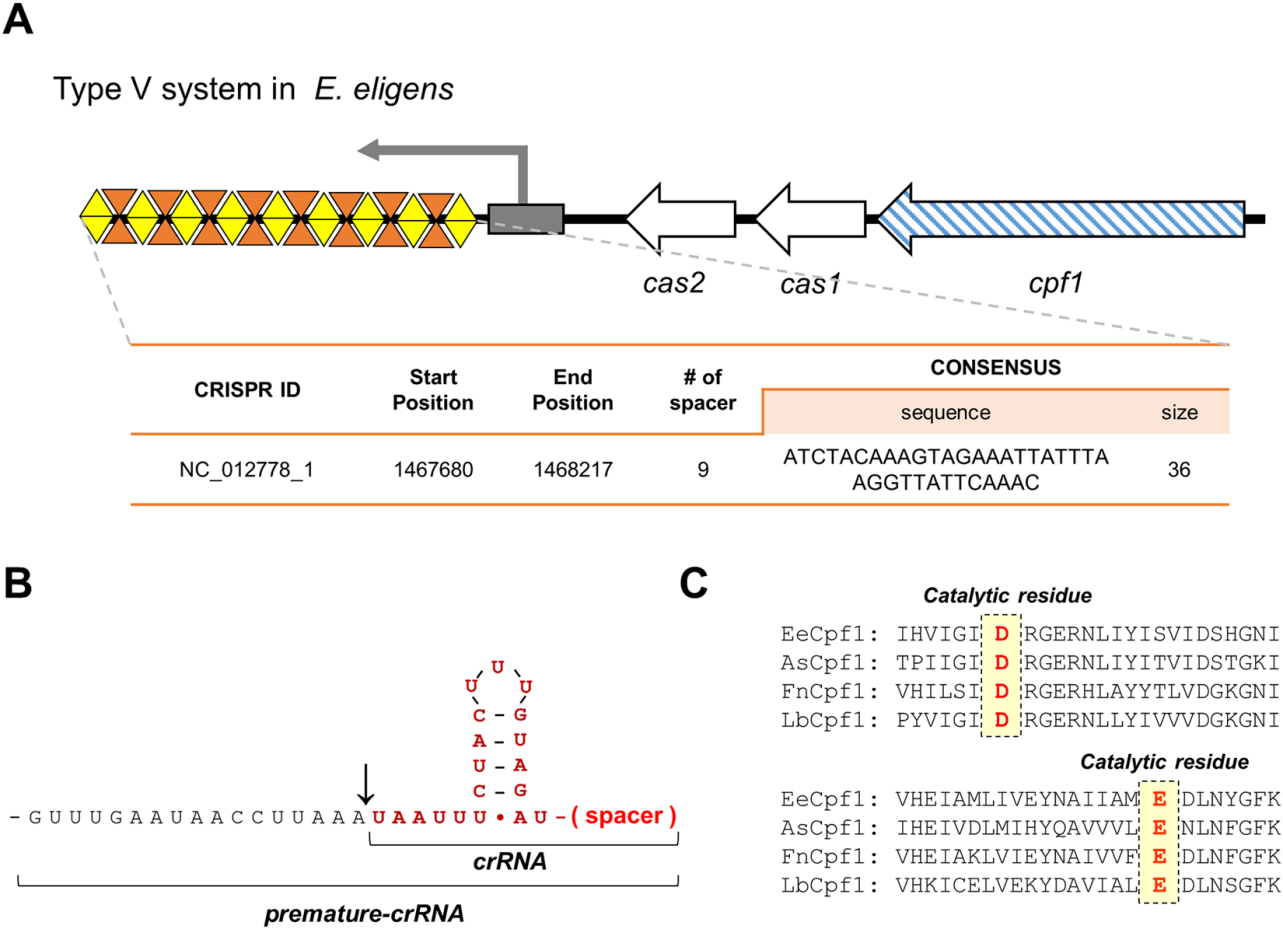
The CRISPR-Cas system of *E*. *eligens*. (**A**) Type V-CRISPR loci were identified within the genome of *E*. *eligens*. The white arrows indicate the ORFs present in the *E*. *eligens* genome, and the blue arrows represent the *cpf1* gene that is specific to type V. (**B**) The predicted secondary structures of the premature CRISPR-RNA of crRNA with a 5′ handle region (red) are shown. (**C**) Multiple sequence alignments of EeCpf1 with the commonly known orthologs of three Cpf1 proteins are shown. The numbering is shown on the right side of the alignment. Red highlights indicate that the catalytic residues are conserved (FnCpf1, *Francisella novicida* U112; AsCpf1, *Acidaminococcus sp*. BV36L; and LbCpf1, *Lachnospiraceae bacterium* ND2006).

The human gut-derived bacterium *Eubacterium eligens* has one CRISPR locus in the circular chromosome (2,144,190 bp) determined by the CRISPR database analysis. The CRISPR locus in *E*. *eligens* contains 36 bp of repeat sequences and 25–29 bp of spacers. When the ORFs near the CRISPR loci were analyzed, the type V system was located next to the *cas1*, *cas2* and *cpf1* (*cas12a)* genes **(**Fig. 1A**)**. Interestingly, the *cas1* gene in *E*. *eligens*, where the protein is expected to be involved in the adaptation stage of the CRISPR system, is significantly smaller than any other *cas1* genes reported so far^16^. The repeat sequences are predicted to form a highly conserved crRNA scaffold in Cpf1 proteins, such as *Fn*Cpf1, *As*Cpf1, and *Lb*Cpf1^17^ **(**Fig. 1B**)**. The conservation of the stem-loop scaffolds indicates that the EeCpf1 protein may recognize the 5′ T-rich PAM sequence according to previous data^11^. Based on sequential alignment with three Cpf1 orthologs, the RuvC domain of EeCpf1 retains two essential catalytic residues (Asp880 and Glu965) that are conserved in the Cpf1 family **(**Fig. 1C**)**. EeCpf1 showed an ~35% sequence homology with the reportedly editable mammalian gene Cpf1s.

### *In vitro* DNA cleavage of EeCpf1

**Figure 2:**
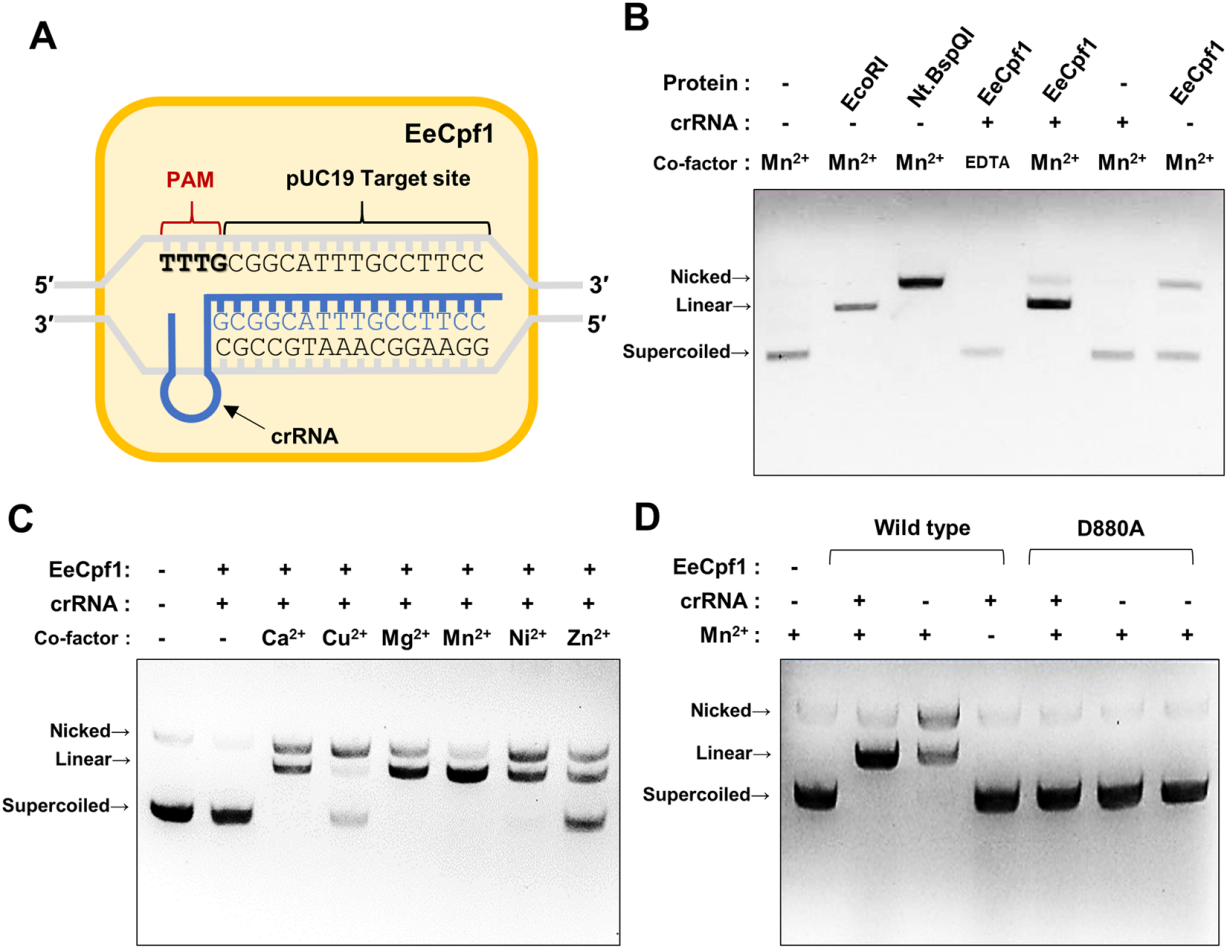
*In vitro* target DNA cleavage activity of EeCpf1. (**A**) Scheme for EeCpf1-crRNA bound to pUC19. The red characters in the pUC19 indicate the PAM motif of EeCpf1. (**B**) The target DNA cleavage activities of EeCpf1 are shown. EeCpf1 (150 nM) in the presence/absence of crRNA (5 mM) was incubated with a target plasmid (pUC19, 200 nM) that contained the TTTN PAM. (**C**) The metal ion-dependent DNase activities are shown. The conditions of the nuclease assays are indicated: Ca^2+^, 1 mM CaCl_2_; Cu^2+^, 1 mM CuSO_4_; Mg^2+^, 1 mM MgCl_2_; Mn^2+^, 1 mM MnCl_2_; Ni^2+^, 1 mM NiCl_2_; Zn^2+^, 1 mM ZnSO_4_. (**D**) DNase activity by the EeCpf1 wild-type and catalytic mutant (D880A) is shown.

To characterize EeCpf1 for its nucleotide cleavage activity, we expressed and purified EeCpf1 proteins from *E*. *coli* and then reconstituted the Cpf1 ribonucleoproteins (RNPs) with *in vitro*-transcribed crRNAs. Previously, an *in vitro* PAM identification assay revealed that the PAM sequence is predominantly T-rich (5′-TTTN-3′) in EeCpf1^18^. We used a double-stranded plasmid (pUC19) bearing the 5′-TTTN-3′ PAM as a DNA substrate and synthesized the crRNA that corresponded to a target in the plasmid **(**Fig. 2A**)**. The *in vitro* DNA cleavage assay showed that EeCpf1 cleaved the target DNA of the plasmid in a crRNA-dependent manner to produce linear DNA **(**Fig. 2B**)**. In the absence of crRNA, EeCpf1 produced a band (lane 7) that migrated with a pattern that corresponded to the pUC19 plasmid that was nicked by *Nt*.*BspQI*; this indicates that EeCpf1 can nick dsDNA in the absence of a crRNA. Since the nuclease activity of Cpf1 is known to be metal-dependent, we further determined the metal ion dependency of EeCpf1. The results showed that the metal ion Mn^2+^, as well as Mg^2+^, Ni^2+^ and Ca^2+^ but not Cu^2+^ or Zn^2+^, enabled EeCpf1 to cleave the target DNA substrate **(**Fig. 2C**)**. We generated an active-site mutant of the RuvC domain that contained a D880A substitution and examined its effect on DNA cleavage activity. The EeCpf1 (D880A) mutant abolished both nick and double-stranded DNA cleavage activity **(**Fig. 2D**)**. These data demonstrate that EeCpf1 shares the same crRNA-mediated DNA cleavage feature as those observed in other Type V systems.

### EeCpf1 can edit the mammalian genomes of mouse cells

**Figure 3:**
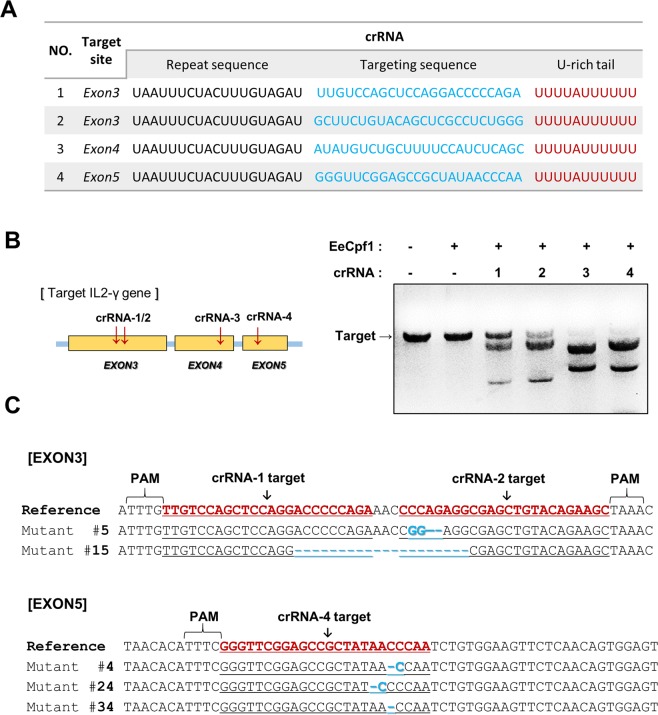
Genome editing by EeCpf1 in mouse embryonic cells. (**A**) Four sites of target crRNA in the IL2R-γ gene are shown. The target recognition region is indicated in blue, and the U-rich tail is indicated in red. (**B**) The *in vitro* DNase activity of EeCpf1 on the IL2R-γ gene. The target gene was generated by PCR and reacted with the EeCpf1-RNP complex (150 nM) at 37 °C. The cleavage sites were mapped to the IL2R-γ gene. The scissors indicate the cleaved sites. (**C**) Sanger sequencing traces from the EeCpf1-digested target gene in blastocysts.

Next, we explored the capacity of the EeCpf1 protein to cleave endogenous genomic loci in mammalian cells. We expressed and purified the human codon-optimized EeCpf1 proteins from *E*. *coli*. Two nuclear localization signals (NLSs) were attached to each N- and C-terminus of EeCpf1 to ensure their nuclear compartmentalization in mammalian cells. The interleukin 2 receptor gamma (IL2R-γ), an essential enzyme in lymphocyte development and one of the candidate genes for the production of immunodeficient mice, was designated as a target. Using Cas-OFFinder and off-target analysis, four sites (two sites in exon 3 and one each in exons 4 and 5) with low sequence homologies to other sequences were selected within the IL2R-γ gene to avoid off-target mutagenesis **(**Supplementary Fig. 1**)**, and the corresponding four crRNAs were designed^19^ **(**Fig. 3A**)**. Previously, the extension of crRNA was reported to enhance the gene editing efficiency of AsCpf1 inside cells^20^. To empower the gene editing efficiency of EeCpf1, we designed each crRNA with the addition of a U-rich tail (U_4_AU_6_) to the 3′-end of the RNA. When the activity of EeCpf1 was measured *in vitro*, four target sites in the IL2R-γ gene that were generated by PCR were all specifically cleaved by the preassembled EeCpf1 RNPs **(**Fig. 3B**)**. Subsequently, we microinjected the recombinant EeCpf1 protein and a mixture of four crRNAs into one-cell-stage embryos, and we cultured the mouse embryos *in vitro* and obtained blastocysts. Sanger sequencing results showed that five out of 35 (15%) blastocysts carried mutations in the IL2R-γ gene. In exon 3, a 20 bp sequence was deleted by overlapping the targets of crRNA1 and/or crRNA2 with a mutation efficiency of 6%. No mutation was found in exon 4 that was generated by crRNA3. The target site in exon 5 showed a 1 bp deletion and a 1 bp change with a 10% efficiency **(**Fig. 3C**)**. The target specificity of EeCpf1 was evaluated for the five genome-wide off-target sites with mismatches ranging from 3- to 10-bp **(**Supplementary Fig. 2A**)**. The results showed no detectable off-target effects in IL2R-γ-mutated blastocysts from the T7E1 assay **(**Supplementary Fig. 2B**)** or Sanger sequencing analyses **(**Supplementary Fig. 3A–C**)**, which is in agreement with the low off-target effects of Cpf1 proteins in mice^19^. Together, the DNA sequencing charts exhibited five kinds of insertion and deletion mutations at the three target sites by EeCpf1 to yield the mutagenesis embryo of the IL2R-γ gene.

**Figure 4:**
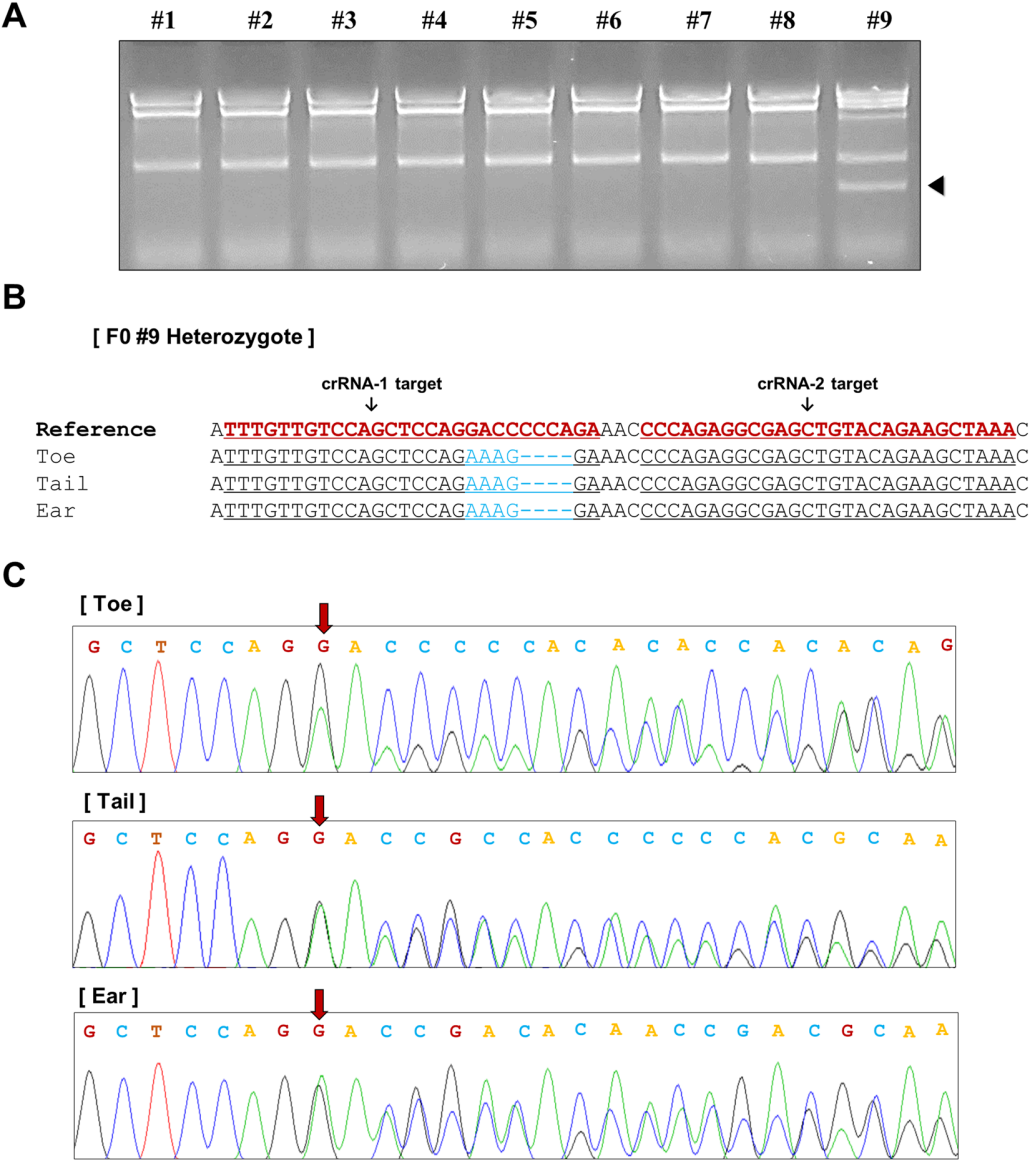
Generation of IL2R-γ knockout mice by EeCpf1-mediated gene targeting. (**A**) T7EI screening of knockout newborns derived from EeCpf1/crRNA injection. (**B**) Sequencing traces of biopsies encompassing the IL2R-γ target region from mutant mouse #9 presented in A. (**C**) Sanger sequencing chromatogram of genomic regions targeted by EeCpf1/crRNA. The red arrows point to overlapping peaks.

To produce IL2R-γ knockout mice, we microinjected a mixture of two crRNAs (target1/target2) and the Eecpf1 protein into 125 one-cell-stage embryos and obtained 76 two-cell-stage embryos (survival rate 60.8%). The 76 surviving embryos were transferred into pseudopregnant C57BL/6J female mice, and nine live animals were born. T7EI-based genotyping analyses identified one mutant (11%) out of nine F0 generation mice **(**Fig. 4A**)**. Sanger sequencing analyses showed that the F0 heterozygote carried mutation sites with 4 bp deletions and 3 bp changes, which were consistently observed in different tissues **(**Fig. 4B**)**, indicating no mosaicism among those three F0 biopsies **(**Fig. 4B,C**)**. These results demonstrate that EeCpf1 could enable genome editing in mammalian cells.

## Discussion

We presented the *in vivo* DNA cleavage activity of Cpf1 from *E*. *eligens* for the first time; we used this activity for gene editing to produce knockout mice. Among the four targeted sites in the IL2R-γ gene, which were all specifically cleaved by the EcCpf1 RNP complex *in vitro*, three sites were successfully mutated at the target loci in the mouse blastocysts, while one site was not mutated at the target loci. We assumed that there may be an epigenetic modification or chromatin structural change around the target region that impaired the accessibility to the target site by EeCpf1^21^. Alternatively, the secondary structure of crRNA3 could have affected the formation of the EeCpf1 RNP complex^22^. Conclusively, the engineering of crRNA by the addition of a U-rich tail to the 3′-end of the RNA and multiple site targeting by crRNAs is an effective method to induce mutagenesis in genome nucleotides by EeCpf1. Recent reports have proposed that the 3′-overhang of the crRNA may have contributed to the effective binding of the RNA to the Cpf1 protein, yielding stable formation of the ribonucleoprotein complexes inside the cells^20^. In addition, the EeCpf1/engineered crRNA RNP complex did not show cytotoxicity or off-target effects in mouse embryos. In this respect, it will be interesting to know whether some Cpf1 orthologs, for which activity was only reported *in vitro*, will exhibit specific *in vivo* DNA cleavage activities if the same method from this study is applied. Notably, the weak activity of FnCpf1 was observed in mammalian cells, whereas FnCpf1 exhibits robust activity in plant cells^23^; this indicates that Cpf1 orthologs may have different activities depending on the organism. Therefore, the availability of additional Cpf1 orthologs with specific target cleavage activities that were presented in this study will further expand the genome editing options for a wide range of organisms. Furthermore, *E*. *eligens* is a common gut Firmicute bacterium and a major contributor to the gut microbiome^24^. Since perturbation of the microbiota and metabolome has been associated with various diseases and metabolic conditions, targeted manipulation of the microbiome by Cpf1 originating from *E*. *eligens* would be one of the potential therapeutic applications of the EcCpf1 protein.

## Supplementary information

Supplementary Information
