# Supplementary material for: In vivo genome editing using the Cpf1 ortholog derived from Eubacterium eligens
Source: Sci Rep. 2019 Sep 26;9:13911. doi: 10.1038/s41598-019-50423-6 (PMC6763456; doi:10.1038/s41598-019-50423-6)

Supplementary Information

In vivo genome editing using the Cpf1 ortholog derived from *Eubacterium eligens*

Woo-Chan Ahn[1,2*], Kwang-Hyun Park[1*], In Seon Bak[3], Hyung-Nam Song[1], An Yan[1], Su-Jin Lee[1,5], Mira Jung[3], Kyeong-Won Yoo[3], Dae-Yeul Yu[4], Yong-Sam Kim[4], Byung-Ha Oh[2] and Eui-Jeon Woo[1,5]**

[1]Disease Target Structure Research Center, Korea Research Institute of Bioscience and Biotechnology (KRIBB), Daejeon 305-806, Republic of Korea

[2]Department of Biological Sciences, KAIST Institute for the Biocentury, Korea Advanced Institute of Science and Technology, Daejeon 305-701, Republic of Korea

[3]Genome Engineering Laboratory, GHBio Inc, Daejeon 305-806, Republic of Korea

[4] Genome Editing Research Center, Korea Research Institute of Bioscience and Biotechnology, (KRIBB), Daejeon 305-806, Republic of Korea

[5]Department of Analytical Bioscience, University of Science and Technology (UST), Daejeon 305-333, Republic of Korea

Supplementary Figure 1 ~ 3

Supplementary Table 1

Uncropped images for scanned gels

Supplementary Figure

sgRNA	Target sequence	Exon	Strand	Off-targets			
				0	1	2	3
1	TTGTCCAGCTCCAGGACCCCCAGA	3	+	0	0	0	1
2	CCCAGAGGCGAGCTGTACAGAAGC	3	-	0	0	0	0
3	GCTGAGATGGAAAAGCAGACATAT	4	-	0	0	0	0
4	GGGTTCGGAGCCGCTATAACCCAA	5	+	0	0	0	0

Supplementary Figure 1. Off-target sequence analysis of EeCpf1/gRNA

Number of potential off-target site in the mouse genome by Cas-OFFinder (http://www.rgenome.net/Cas-Offinder).

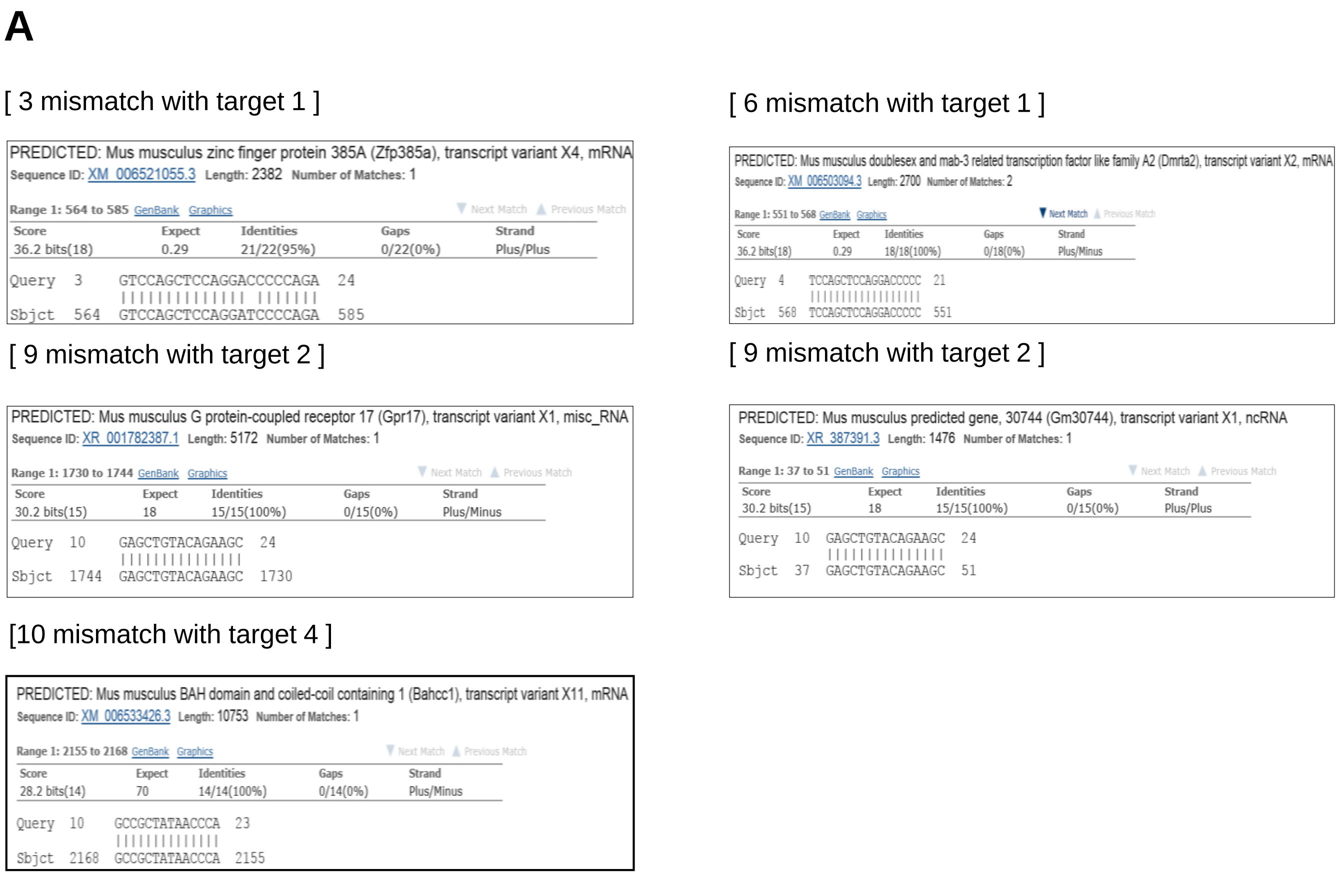

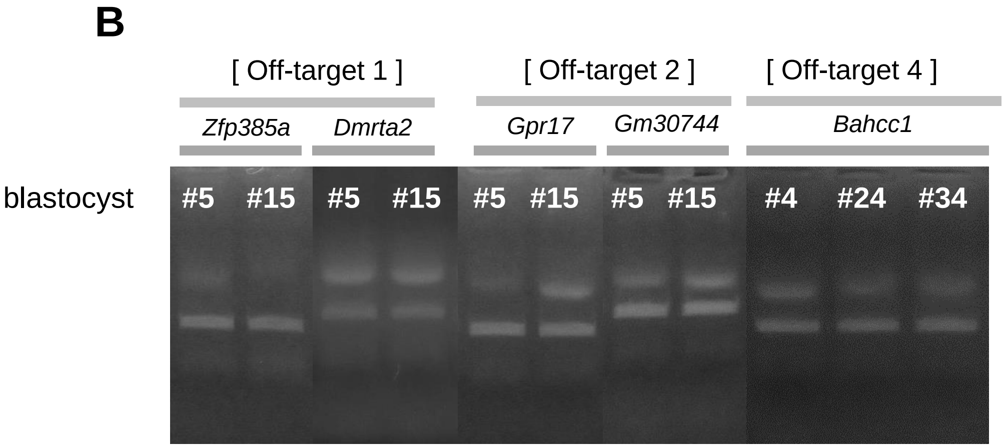

Supplementary Figure 2. **Analysis of Off-target effects in IL2R-γ-edited blastocysts**

(A) The alignment of the off-target sites with the targeting sequence. Mismatched (on-target site vs off-target site) nucleotides and the targeting sequence number of gRNAs are indicated.

(B) T7EI assay of the potential off-target site in edited blastocysts. Bands were shown in PCR amplicons from blastocysts injected with the EeCpf1/crRNA mixtures. White letters indicate the numbers of mutated blastocysts.

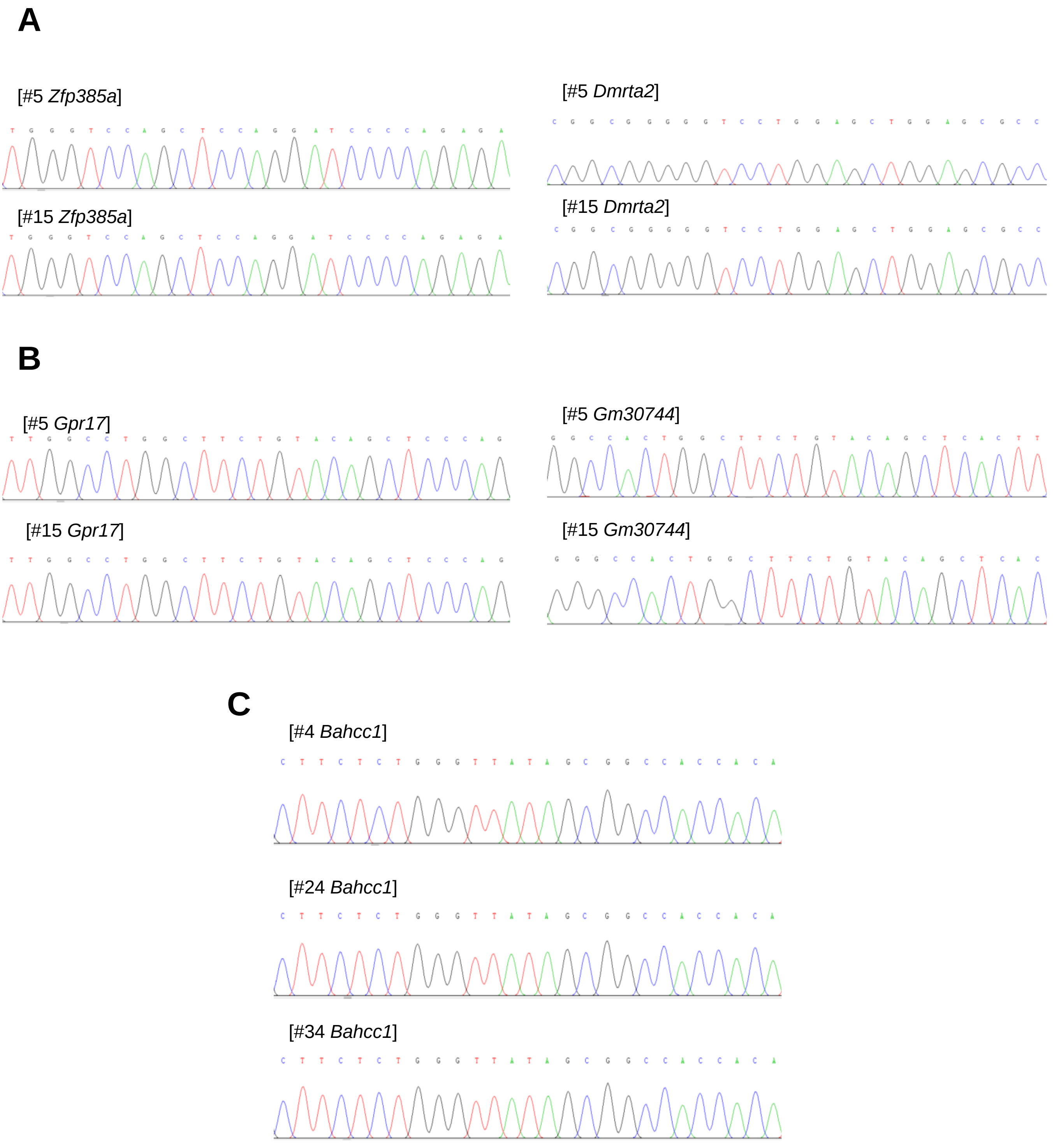

Supplementary Figure 3. Sanger sequencing of potential off-target sites in EeCapf1-edited blastocysts

(A) Chromatogram of the Sanger sequence for 2 potential off-target sites by gRNA-1.

(B) Chromatogram of the Sanger sequence for 2 potential off-target sites by gRNA-2.

(C) Chromatogram of the Sanger sequence for 3 potential off-target sites by gRNA-4.

Supplementary Table 1. DNA sequence

- **Protein ; EeCpf1-CPD-6xhis-tag**

ATGAATGGAAATCGTAGCATAGTTTACAGAGAGTTTGTGGGGGTTATACCAGTAGCAAAGACACTAAGGAATGAATTAAGACCTGTTGGGCATACACAGGAGCATATAATACAGA
ATGGACTTATCCAGGAAGATGAGTTAAGACAAGAGAAAAGCACAGAGTTAAAAAATATAATGGATGATTATTATAGAGAATATATAGATAAATCATTATCAGGTGTAACTGATCT
AGATTTTACTCTGCTTTTTGAATTAATGAATTTGGTTCAGTCAAGTCCATCAAAAGATAATAAGAAAGCATTAGAAAAAGAGCAGAGCAAAATGAGAGAACAGATATGCACACAT
CTGCAATCAGATTCTAATTACAAAAATATATTTAACGCAAAGCTTTTGAAAGAGATACTGCCAGATTTTATTAAAAATTATAATCAATATGATGTTAAAGACAAAGCTGGCAAAC
TGGAAACATTAGCATTGTTTAATGGTTTTAGCACATATTTTACGGATTTTTTTGAAAAGAGAAAAAATGTATTTACTAAAGAGGCTGTTTCTACATCTATAGCTTATAGAATTGT
GCATGAAAATTCTTTGATTTTTCTTGCTAATATGACATCATATAAGAAAATCTCAGAAAAGGCATTGGATGAAATAGAAGTAATTGAAAAAAATAATCAGGATAAAATGGGTGAT
TGGGAATTGAATCAGATATTTAATCCGGATTTTTATAACATGGTATTAATTCAGTCAGGAATAGACTTTTACAATGAAATATGTGGTGTGGTTAATGCACATATGAATCTTTATT
GCCAGCAGACAAAGAATAATTACAATTTATTCAAAATGCGTAAGCTGCATAAGCAAATCCTTGCGTATACATCAACTTCATTTGAAGTACCTAAAATGTTTGAAGATGATATGAG
CGTATATAATGCTGTTAATGCGTTTATTGATGAAACAGAAAAAGGCAATATAATTGGAAAACTGAAAGATATCGTTAATAAATATGATGAATTAGATGAGAAAAGAATATATATA
TCAAAAGATTTTTATGAGACATTATCATGTTTTATGAGTGGTAACTGGAATTTAATTACAGGTTGCGTTGAAAATTTTTATGATGAAAATATACATGCAAAAGGTAAGTCTAAAG
AAGAAAAAGTTAAAAAAGCTGTAAAAGAAGATAAATATAAGAGTATTAATGATGTTAATGATTTAGTTGAGAAATATATAGATGAAAAAGAACGAAATGAATTTAAAAATTCTAA
TGCTAAGCAATATATAAGGGAAATTAGTAATATAATAACTGATACAGAAACAGCACATTTGGAATACGATGATCATATAAGTCTGATTGAGAGCGAAGAAAAAGCTGATGAAATG
AAGAAAAGGCTTGATATGTATATGAACATGTATCATTGGGCAAAGGCATTTATTGTTGATGAAGTACTGGATAGAGATGAAATGTTTTATTCAGATATAGACGATATCTATAATA
TATTAGAAAATATAGTGCCATTATATAACAGGGTGAGAAATTATGTTACACAGAAACCATACAATTCTAAGAAAATAAAACTTAATTTTCAAAGTCCTACACTTGCTAATGGTTG
GTCGCAGAGTAAAGAGTTTGATAATAATGCAATAATTTTAATAAGAGATAATAAGTATTATCTGGCAATTTTTAATGCTAAAAATAAACCCGATAAAAAGATTATACAGGGAAAT
TCTGATAAAAAGAACGATAATGATTATAAGAAGATGGTATATAATCTGTTGCCGGGGGCGAATAAAATGCTTCCTAAAGTGTTCTTATCAAAGAAAGGAATAGAAACATTTAAGC
CATCTGATTATATAATTTCCGGTTATAATGCACACAAACATATTAAGACAAGTGAGAATTTTGATATCAGTTTTTGTAGGGATTTAATTGATTATTTTAAGAACAGCATAGAGAA
ACATGCGGAATGGAGAAAATATGAATTTAAATTCTCTGCAACTGATAGTTATAGCGATATAAGTGAATTCTACCGTGAAGTTGAAATGCAGGGGTATAGAATTGACTGGACTTAT
ATAAGTGAAGCTGATATTAATAAGCTTGATGAGGAAGGCAAGATATATTTATTTCAGATTTATAATAAGGACTTCGCAGAGAACAGTACAGGAAAAGAAAATCTTCACACTATGT
ATTTCAAGAACATTTTTAGTGAAGAAAATCTTAAGGATATCATTATTAAATTAAATGGACAGGCTGAATTGTTTTACAGAAGGGCAAGCGTAAAAAATCCAGTTAAGCATAAAAA
AGATTCTGTATTAGTTAATAAGACATATAAAAATCAGCTTGATAATGGTGATGTTGTAAGAATACCGATTCCAGATGATATATATAATGAAATTTATAAAATGTATAATGGATAT
ATTAAAGAAAGTGACTTGTCAGAAGCTGCAAAGGAATATCTTGATAAAGTAGAGGTCAGGACTGCCCAAAAGGATATTGTTAAAGATTACAGATATACAGTTGATAAATATTTTA
TACATACGCCAATTACTATTAACTATAAAGTGACAGCACGAAATAATGTAAATGATATGGTTGTAAAATATATAGCACAAAATGATGATATACATGTTATTGGAATTGACAGGGG
TGAGCGTAATCTTATATATATTTCTGTTATAGATTCTCATGGAAATATAGTTAAACAGAAAAGTTATAATATATTGAATAATTATGATTATAAGAAGAAGCTGGTTGAGAAGGAA
AAGACCAGAGAATATGCGAGAAAGAACTGGAAATCTATAGGAAATATTAAAGAGTTGAAAGAAGGATATATTTCGGGGGTAGTTCATGAGATAGCAATGCTTATAGTTGAGTATA
ATGCAATAATTGCTATGGAAGATTTGAATTATGGATTTAAGCGAGGTCGTTTTAAAGTTGAACGACAGGTATACCAGAAGTTCGAGAGTATGCTTATTAATAAGCTGAATTATTT
TGCTTCTAAAGAAAAATCTGTAGATGAACCAGGGGGACTGTTAAAAGGATATCAATTGACATATGTACCAGATAATATAAAAAATCTTGGGAAACAATGTGGAGTAATATTTTAT
GTACCTGCTGCATTTACATCAAAAATAGATCCGTCAACCGGATTTATCAGTGCATTTAATTTTAAGAGCATTTCAACAAATGCTTCAAGAAAACAGTTCTTTATGCAATTTGATG
AAATAAGATATTGTGCCGAAAAAGACATGTTCAGTTTCGGATTTGATTATAATAATTTTGATACCTACAACATTACCATGGGTAAGACACAATGGACAGTATATACCAATGGTGA
GAGACTGCAGAGTGAATTCAACAATGCAAGGAGAACAGGCAAAACAAAATCAATAAATCTTACAGAAACAATAAAATTATTATTAGAAGATAATGAAATTAATTATGCTGATGGA
CATGATATAAGGATAGATATGGAGAAGATGGATGAAGATAAAAAGTCTGAATTCTTTGCACAACTGTTATCTTTATATAAACTTACAGTTCAGATGAGAAACAGTTATACTGAAG
CCGAGGAACAGGAGAATGGAATAAGTTACGATAAAATAATATCACCTGTAATCAATGATGAAGGAGAATTTTTTGACTCAGATAATTATAAAGAATCAGATGATAAAGAATGCAA
GATGCCAAAAGATGCAGATGCAAATGGTGCATATTGTATAGCGCTTAAGGGATTATATGAGGTGTTAAAGATAAAGTCTGAGTGGACAGAAGATGGTTTTGACAGGAACTGCCTG
AAATTACCTCATGCTGAATGGTTGGATTTTATACAGAATAAAAGGTATGAAGTCGACAAGCTTGCATTAGCGGATGGAAAAATACTCCATAATCAAAATGTTAATAGCTGGGGCC
CGATTACGGTTACACCAACGACAGATGGTGGTGAAACCCGCTTCGACGGTCAAATCATCGTTCAAATGGAAAACGACCCGGTAGTAGCAAAAGCGGCAGCCAATTTAGCAGGTAA
ACATGCTGAAAGCAGTGTGGTGGTGCAGCTCGATTCAGACGGCAACTATCGCGTGGTGTATGGCGATCCGTCAAAACTGGATGGAAAGCTACGTTGGCAGTTGGTGGGGCATGGT
CGCGACCACTCAGAAACTAACAATACTCGCTTAAGTGGTTACAGTGCCGATGAGTTGGCCGTGAAATTGGCCAAGTTCCAACAGTCGTTTAATCAAGCCGAAAACATCAACAACA
AACCGGATCACATCAGTATTGTTGGTTGTTCTTTGGTGAGTGACGACAAGCAAAAAGGCTTTGGTCATCAGTTTATTAACGCGATGGATGCGAATGGTCTTCGTGTCGATGTCTC
TGTTCGTAGTTCTGAACTGGCCGTAGACGAGGCGGGACGTAAGCATACCAAGGACGCGAATGGCGATTGGGTTCAAAAGGCAGAAAACAACAAAGTTTCGCTAAGCTGGGACGCG
CAAGGTCTCGAGCACCACCACCACCACCACTGA

- **Protein ; EeCpf1-NLS-CPD-6xhis-tag**

ATGAACGGCAATAGGTCCATCGTGTACCGCGAGTTCGTGGGCGTGATCCCCGTGGCCAAGACCCTGAGGAATGAGCTGCGCCCTGTGGGCCACACACAGGAGCACATCATCCAGA
ACGGCCTGATCCAGGAGGACGAGCTGCGGCAGGAGAAGAGCACCGAGCTGAAGAACATCATGGACGATTACTATAGAGAGTACATCGATAAGTCTCTGAGCGGCGTGACCGACCT
GGACTTCACCCTGCTGTTCGAGCTGATGAACCTGGTGCAGAGCTCCCCCTCCAAGGACAATAAGAAGGCCCTGGAGAAGGAGCAGTCTAAGATGAGGGAGCAGATCTGCACCCAC
CTGCAGTCCGACTCTAACTACAAGAATATCTTTAACGCCAAGCTGCTGAAGGAGATCCTGCCTGATTTCATCAAGAACTACAATCAGTATGACGTGAAGGATAAGGCCGGCAAGC
TGGAGACACTGGCCCTGTTTAATGGCTTCAGCACATACTTTACCGACTTCTTTGAGAAGAGGAAGAACGTGTTCACCAAGGAGGCCGTGAGCACATCCATCGCCTACCGCATCGT
GCACGAGAACTCCCTGATCTTCCTGGCCAATATGACCTCTTATAAGAAGATCAGCGAGAAGGCCCTGGATGAGATCGAAGTGATCGAGAAGAACAATCAGGACAAGATGGGCGAT
TGGGAGCTGAATCAGATCTTTAACCCTGACTTCTACAATATGGTGCTGATCCAGTCCGGCATCGACTTCTACAACGAGATCTGCGGCGTGGTGAATGCCCACATGAACCTGTACT
GTCAGCAGACCAAGAACAATTATAACCTGTTCAAGATGCGGAAGCTGCACAAGCAGATCCTGGCCTACACCAGCACCAGCTTCGAGGTGCCCAAGATGTTCGAGGACGATATGAG
CGTGTATAACGCCGTGAACGCCTTCATCGACGAGACAGAGAAGGGCAACATCATCGGCAAGCTGAAGGATATCGTGAATAAGTACGACGAGCTGGATGAGAAGAGAATCTATATC
AGCAAGGACTTTTACGAGACACTGAGCTGCTTCATGTCCGGCAACTGGAATCTGATCACAGGCTGCGTGGAGAACTTCTACGATGAGAACATCCACGCCAAGGGCAAGTCCAAGG
AGGAGAAGGTGAAGAAGGCCGTGAAGGAGGACAAGTACAAGTCTATCAATGACGTGAACGATCTGGTGGAGAAGTATATCGATGAGAAGGAGAGGAATGAGTTCAAGAACAGCAA
TGCCAAGCAGTACATCCGCGAGATCTCCAACATCATCACCGACACAGAGACAGCCCACCTGGAGTATGACGATCACATCTCTCTGATCGAGAGCGAGGAGAAGGCCGACGAGATG
AAGAAGCGGCTGGATATGTATATGAACATGTACCACTGGGCCAAGGCCTTTATCGTGGACGAGGTGCTGGACAGAGATGAGATGTTCTACAGCGATATCGACGATATCTATAATA
TCCTGGAGAACATCGTGCCACTGTATAATCGGGTGAGAAACTACGTGACCCAGAAGCCCTACAACTCTAAGAAGATCAAGCTGAATTTCCAGAGCCCTACACTGGCCAATGGCTG
GTCCCAGTCTAAGGAGTTCGACAACAATGCCATCATCCTGATCAGAGATAACAAGTACTATCTGGCCATCTTCAATGCCAAGAACAAGCCAGACAAGAAGATCATCCAGGGCAAC
TCCGATAAGAAGAACGACAACGATTACAAGAAGATGGTGTATAACCTGCTGCCAGGCGCCAACAAGATGCTGCCCAAGGTGTTTCTGTCTAAGAAGGGCATCGAGACATTCAAGC
CCTCCGACTATATCATCTCTGGCTACAACGCCCACAAGCACATCAAGACAAGCGAGAATTTTGATATCTCCTTCTGTCGGGACCTGATCGATTACTTCAAGAACAGCATCGAGAA
GCACGCCGAGTGGAGAAAGTATGAGTTCAAGTTTTCCGCCACCGACAGCTACTCCGATATCTCTGAGTTCTATCGGGAGGTGGAGATGCAGGGCTACAGAATCGACTGGACATAT
ATCAGCGAGGCCGACATCAACAAGCTGGATGAGGAGGGCAAGATCTATCTGTTTCAGATCTACAATAAGGATTTCGCCGAGAACAGCACCGGCAAGGAGAATCTGCACACAATGT
ACTTTAAGAACATCTTCTCCGAGGAGAATCTGAAGGACATCATCATCAAGCTGAACGGCCAGGCCGAGCTGTTTTATCGGAGAGCCTCTGTGAAGAATCCCGTGAAGCACAAGAA
GGATAGCGTGCTGGTGAACAAGACCTACAAGAATCAGCTGGACAACGGCGACGTGGTGAGAATCCCCATCCCTGACGATATCTATAACGAGATCTACAAGATGTATAATGGCTAC
ATCAAGGAGTCCGACCTGTCTGAGGCCGCCAAGGAGTACCTGGATAAGGTGGAGGTGAGGACCGCCCAGAAGGACATCGTGAAGGATTACCGCTATACAGTGGACAAGTACTTCA
TCCACACACCTATCACCATCAACTATAAGGTGACCGCCCGCAACAATGTGAATGATATGGTGGTGAAGTACATCGCCCAGAACGACGATATCCACGTGATCGGCATCGACCGGGG
CGAGAGAAACCTGATCTACATCTCCGTGATCGATTCTCACGGCAACATCGTGAAGCAGAAATCCTACAACATCCTGAACAACTACGACTACAAGAAGAAGCTGGTGGAGAAGGAG
AAAACCCGGGAGTACGCCAGAAAGAACTGGAAGAGCATCGGCAATATCAAGGAGCTGAAGGAGGGCTATATCTCCGGCGTGGTGCACGAGATCGCCATGCTGATCGTGGAGTACA
ACGCCATCATCGCCATGGAGGACCTGAATTATGGCTTTAAGAGGGGCCGCTTCAAGGTGGAGCGGCAGGTGTACCAGAAGTTTGAGAGCATGCTGATCAATAAGCTGAACTATTT
CGCCAGCAAGGAGAAGTCCGTGGACGAGCCAGGAGGCCTGCTGAAGGGCTATCAGCTGACCTACGTGCCCGATAATATCAAGAACCTGGGCAAGCAGTGCGGCGTGATCTTTTAC
GTGCCTGCCGCCTTCACCAGCAAGATCGACCCATCCACAGGCTTTATCTCTGCCTTCAACTTTAAGTCTATCAGCACAAATGCCTCTCGGAAGCAGTTCTTTATGCAGTTTGACG
AGATCAGATACTGTGCCGAGAAGGATATGTTCAGCTTTGGCTTCGACTACAACAACTTCGATACCTACAACATCACAATGGGCAAGACACAGTGGACCGTGTATACAAACGGCGA
GAGACTGCAGTCTGAGTTCAACAATGCCAGGCGCACCGGCAAGACAAAGAGCATCAATCTGACAGAGACAATCAAGCTGCTGCTGGAGGACAATGAGATCAACTACGCCGACGGC
CACGATATCAGGATCGATATGGAGAAGATGGACGAGGATAAGAAGAGCGAGTTCTTTGCCCAGCTGCTGAGCCTGTATAAGCTGACCGTGCAGATGCGCAATTCCTATACAGAGG
CCGAGGAGCAGGAGAACGGCATCTCTTACGACAAGATCATCAGCCCTGTGATCAATGATGAGGGCGAGTTCTTTGACTCCGATAACTATAAGGAGTCTGACGATAAGGAGTGCAA
GATGCCAAAGGACGCCGATGCCAACGGCGCCTACTGTATCGCCCTGAAGGGCCTGTATGAGGTGCTGAAGATCAAGAGCGAGTGGACCGAGGACGGCTTTGATAGGAATTGCCTG
AAGCTGCCACACGCAGAGTGGCTGGACTTCATCCAGAACAAGCGGTACGAGAAAAGGCCGGCGGCCACGAAAAAGGCCGGCCAGGCAAAAAAGAAAAAGGGATCCTACCCATACG
ATGTTCCAGATTACGCTTATCCCTACGACGTGCCTGATTATGCATACCCATATGATGTCCCCGACTATGCCGCATTAGCGGATGGAAAAATACTCCATAATCAAAATGTTAATAG
CTGGGGCCCGATTACGGTTACACCAACGACAGATGGTGGTGAAACCCGCTTCGACGGTCAAATCATCGTTCAAATGGAAAACGACCCGGTAGTAGCAAAAGCGGCAGCCAATTTA
GCAGGTAAACATGCTGAAAGCAGTGTGGTGGTGCAGCTCGATTCAGACGGCAACTATCGCGTGGTGTATGGCGATCCGTCAAAACTGGATGGAAAGCTACGTTGGCAGTTGGTGG
GGCATGGTCGCGACCACTCAGAAACTAACAATACTCGCTTAAGTGGTTACAGTGCCGATGAGTTGGCCGTGAAATTGGCCAAGTTCCAACAGTCGTTTAATCAAGCCGAAAACAT
CAACAACAAACCGGATCACATCAGTATTGTTGGTTGTTCTTTGGTGAGTGACGACAAGCAAAAAGGCTTTGGTCATCAGTTTATTAACGCGATGGATGCGAATGGTCTTCGTGTC
GATGTCTCTGTTCGTAGTTCTGAACTGGCCGTAGACGAGGCGGGACGTAAGCATACCAAGGACGCGAATGGCGATTGGGTTCAAAAGGCAGAAAACAACAAAGTTTCGCTAAGCT
GGGACGCGCAAGGTCTCGAGCACCACCACCACCACCACTGA

Uncropped images for scanned gels.

Figure 2B

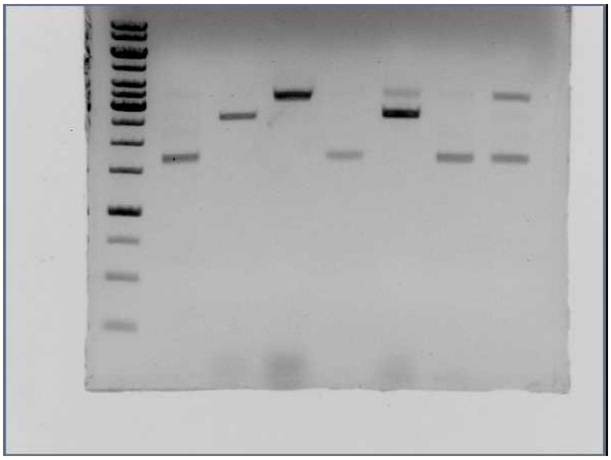

Figure 2C

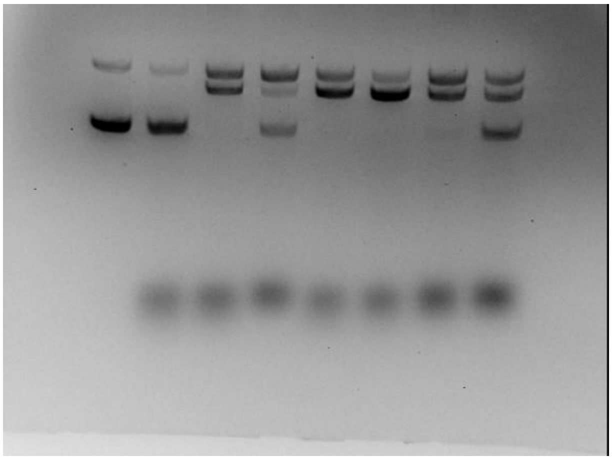

Figure 2D

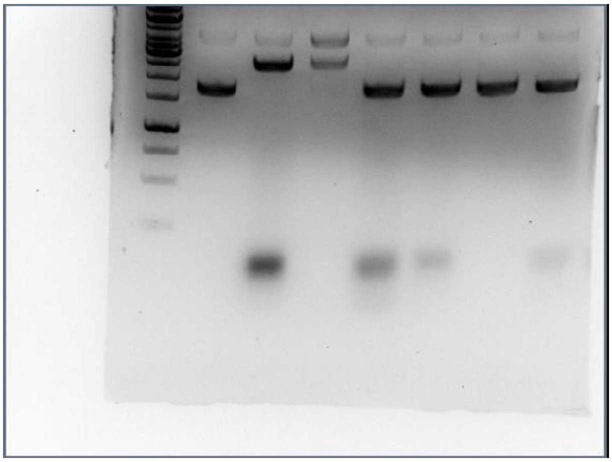

Figure 3B

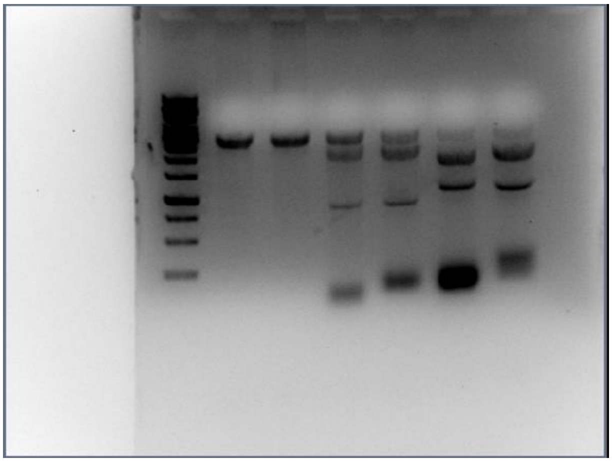

Supplement: Supplementary file 1 — Supplementary Information [file 41598_2019_50423_MOESM1_ESM.pdf]
